# Effect of Bi Content on the Microstructure, Mechanical and Tribological Properties of Cu-Sn Alloy

**DOI:** 10.3390/ma16206658

**Published:** 2023-10-11

**Authors:** Zhenhua Shi, Hong Xu, Guowei Zhang, Yijun Liu, Xiaoyan Ren

**Affiliations:** 1 School of Materials Science and Engineering, North University of China, Taiyuan 030051, China; szh1564067917@163.com (Z.S.);; 2 Department of Mechanical Engineering, Taiyuan Institute of Technology, Taiyuan 030008, China

**Keywords:** Cu-Sn alloy, Cu-Bi-Sn alloy, mechanical property, tribological property

## Abstract

To reduce the use of the toxic Pb element in the Cu-Sn alloy with high friction performance, Cu-xBi-10Sn alloys with different Bi contents were prepared by gravity casting, and the effect of Bi content on the microstructure, mechanical properties and wear property of Cu-Sn alloys were studied. The results showed that the Bi element was distributed in bands or long strips on the dendritic arms and did not form compounds with other elements. With the increase in Bi content, the hardness and tensile strength of Cu-xBi-10Sn alloys present a trend of increasing first and then decreasing. When the Bi content was 7 wt.%, the maximum hardness value was obtained, and the ultimate tensile strength was close to that of Cu-10Pb-10Sn alloy. Compared with Cu-10Pb-10Sn alloy, Cu-7Bi-10Sn alloy also possessed better friction reduction and wear resistance under the oil lubrication condition.

## 1. Introduction

As an excellent friction-reduction material, lead–tin bronze (Pb > 5 wt.%), with high strength, high hardness and good wear resistance, is widely used in bimetallic cylinder blocks, bearings, axial shingles, piston pump rotors and other components that require tribological performance [1,2,3,4,5]. Pb exists commonly as a free state on the α-Cu matrix; however, a layer of Pb film will be formed on the worn surface under the friction condition due to the frictional heat production. This leads to the increase in the material’s boundary lubrication ability and the resistance to friction of the contact point, as well as the improved antiadhesion and wear resistance of alloys. Thus, Pb is currently an irreplaceable material for high-power engines and internal combustion engine components [6,7,8].

However, Pb is a heavily polluting metal and is extremely harmful to humans [9,10,11]. At present, the newest round of equipment iteration has put forward higher requirements for tin bronze friction-reduction materials. With the increase in load in actual working conditions, the wear problem results in premature failure. Therefore, lead-free, high-strength, wear-resistant tin bronze materials have become the trend of the future [12,13]. Bi is a non-toxic and soft metal, and, increasingly, researchers in recent years [14,15,16,17,18] have proposed using the powder metallurgy technique to replace Pb with Bi. It found that the precipitation of Bi to the alloy surface during the friction process plays a good friction reduction and antiadhesion role. At the present stage, bismuth–tin bronze materials fabricated by powder metallurgy have poor densification and are prone to specific gravity segregations of Bi at grain boundaries, resulting in reducing hardness, tensile strength and impact toughness, further leading to the shedding of Bi from the worn surface during the wear process. Using the gravity casting method can solve the above problems, and the casting technique is not restricted by the type of material. Furthermore, this method has more forming advantages for complex components. However, little research has been reported on the bismuth–tin bronze alloy prepared by the casting method in recent years.

Based on the above, in this study, Cu-xBi-10Sn alloys were prepared by using the gravity casting technique, and the effect of Bi content on the microstructure and mechanical properties of tin bronze alloys was studied. Tribological performance of Cu-xBi-10Sn alloy was also examined under oil conditions and the tribological behavior of Bi in tin bronze was further revealed. This work is expected to provide the theoretical guidance for the future application of new antifriction copper alloys, which could replace the traditional lead–tin bronze alloy.

## 2. Materials and Methods

### 2.1. Preparation for Alloys

Cu-xBi-10Sn (x = 3, 5, 7, 10 wt.%) alloys were prepared by the gravity casting technique, in which the metal mold was used. The test was conducted using a pit-type resistance furnace (SXW-18-13, Shanghai, China) for melting. The melting temperature of the copper alloy was 1200 ± 20 °C, the time interval for adding each alloying element was 5 min and the stirring time was 30 s. The preheating temperature of the mold was 200 ± 20 °C. During casting, the melt was poured into the Y-type metal mold, and then the mold was opened after cooling. The chemical compositions of each alloy are shown in Table 1.

**Figure 1:**
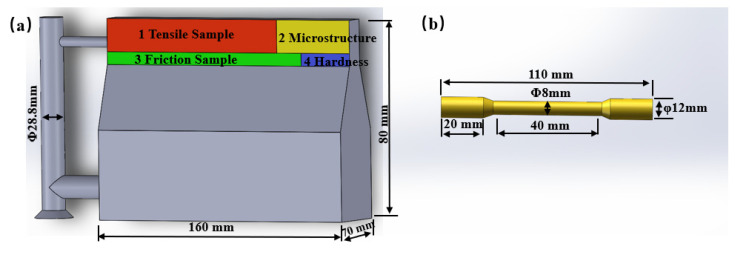
(**a**) Copper alloy ingot and sampling method, (**b**) schematic of the tensile samples.

The casted ingot dimensions and the sampling method for samples are shown in Figure 1a. The red area 1 is the sampling position for the tensile test, which was processed into the tensile samples as depicted in Figure 1b. The samples for microstructure analysis were cut from area 2, the sample at area 3 was used to the friction test, while area 4 was the sampling position for the hardness analysis.

### 2.2. Microstructure Characterization

The specimens were eroded with an etching solution (ammonia water: hydrogen peroxide:water = 1:1:3). The microstructure was observed by 7900 F thermal field emission scanning electron microscope equipped with energy dispersive spectroscopy (EDS) (Bruker, Karlsruhe, German). The phase structure of the alloys was analyzed by X-ray diffractometer (XRD) (Rigaku, Yamanashi, Japan) with Cu Kα radiation in the range of 2*θ* = 10–90°at a scanning rate of 4°/min.

### 2.3. Properties

The tensile test was carried out using WDW-20/30 universal testing machine (SUNS, Shenzhen, China) at a tensile speed of 2.4 mm/min, and the hardness of the alloys was measured using a 5 mm indenter on a HB-300B Brinell hardness tester (Huayin, Laizhou, China) under a load of 250 kgf and holding time of 30 s, and the same specimen was measured five times to take the average value as the final result.

**Figure 2:**
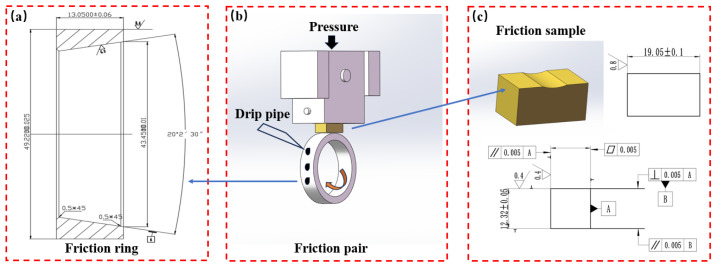
(**a**) Friction ring size, (**b**) schematic diagram of the friction pair, (**c**) friction sample size.

The friction test was conducted on the MRH-3A high-speed ring block wear tester (Yihua, Jinan, China). The friction ring material was 1045 steel, and the size is shown in Figure 2a. The friction pair contact mode is presented in Figure 2b, and the friction sample size is depicted in Figure 2c. The wear test temperature was room temperature, and the test condition was the use of SAE 15W-40 oil lubrication. Before the test, the specimen was cleaned with acetone and weighed with a one ten-thousandth electronic analytical balance to determine the weight before and after the wear. During the test, the friction block was first fixed and then the friction ring was rotated. The speed of 1500 r/min and the load of 250 N were used, and the wear time was 60 min. The weight loss was measured five times to take the average value and then calculate the wear rate. The same test was repeated 3 times, and the average value was the final result. The worn surface was examined by a three-dimensional topography measuring instrument (ER230, Shenzhen, China).

## 3. Results and Discussion

### 3.1. Microstructure

**Figure 3:**
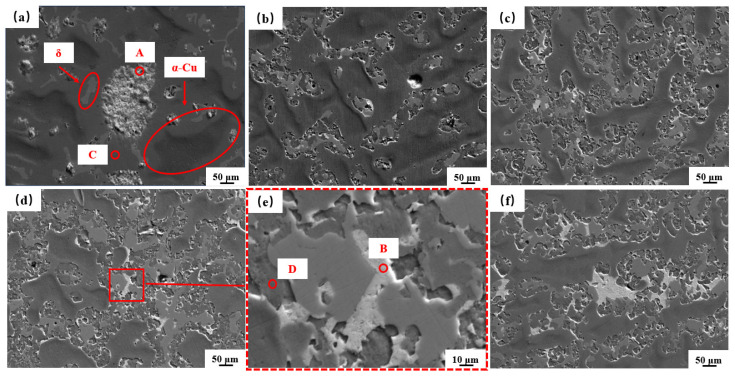
SEM image of the alloys: (**a**) Cu-10Pb-10Sn, (**b**) Cu-3Bi-10Sn, (**c**) Cu-5Bi-10Sn, (**d**) Cu-7Bi-10Sn, (**e**) the enlargement of Cu-7Bi-10Sn alloy and (**f**) Cu-10Bi-10Sn.

Micrographs of Cu-10Pb-10Sn and Cu-xBi-10Sn alloys are shown in Figure 3. The dark gray phase is the α-Cu matrix, the phase with light gray contrast is the δ-phase [19]. From Figure 3a, it can be seen that the white morphology is granular and exists as a free state on the matrix. From Figure 3b–f, it is observed that the white morphology becomes progressively larger and more numerous with increasing Bi content. EDS characterization was carried out to analyze the composition of points A, B, C and D, as shown in Figure 3a,e, and XRD was carried out for the Cu-10Pb-10Sn alloy and the Cu-7Bi-10Sn alloy.

**Figure 4:**
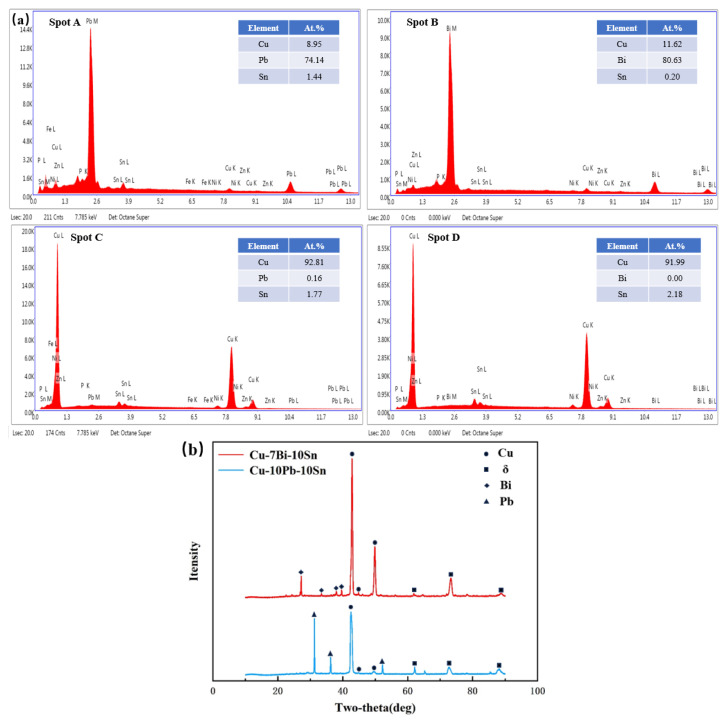
(**a**) EDS results of spot A, spot B, spot C and spot D in Figure 3, (**b**) XRD results of the alloys.

EDS and XRD results are shown in Figure 4a,b, respectively. Figure 4a illustrates that the white phase with granular morphology in is the Pb phase, and the striped one corresponds to the Bi phase. According to the scanning results of points C and D, it can be seen that the α-Cu phase does not contain dissolved Pb or Bi. From Figure 4b, α-Cu phase, δ-phase, Pb phase and Bi phase are detected in the alloy [20,21,22], and no other phases containing the Bi element are observed. Among these phases, the α-Cu phase is the main phase of Cu-Sn alloy, and the δ-phase is the hard phase present between the dendrites of the matrix which increases the strength and hardness of the alloy. The Pb and Bi phases are soft phases and they are distributed on the matrix. It is informing that both Pb and Bi do not react with the copper matrix to form compounds. However, there is still a difference in the distribution location and morphology on the matrix with respect to Pb and Bi phases. Pb particles are distributed independently on the matrix in a granular structure, whereas Bi particles are distributed around the dendrites and the δ-phase in the form of bands.

**Figure 5:**
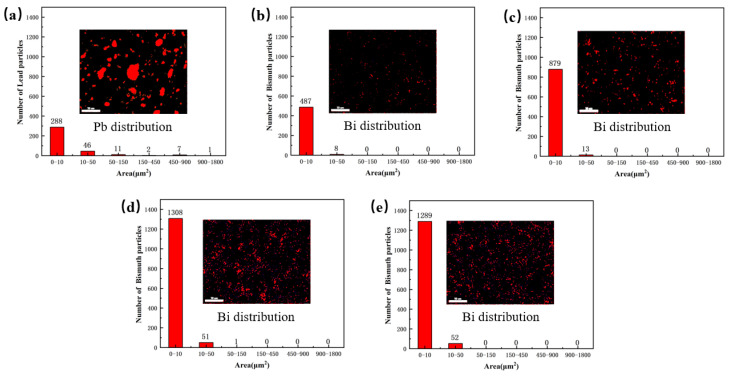
Statistics of Pb and Bi particles in alloys: (**a**) Cu-10Pb-10Sn, (**b**) Cu-3Bi-10Sn, (**c**) Cu-5Bi-10Sn, (**d**) Cu-7Bi-10Sn and (**e**) Cu-10Bi-10Sn.

The Pb and Bi particles in the α-Cu matrix were calculated by using Image J software (version 1.48), and the results are presented in Figure 5. A total of the particles in the range of 0–1800 μm^2^ were counted. From Figure 5a, the size of the Pb particle region is concentrated within 900 μm^2^, among which the number of Pb particles in the range of 0–50 μm^2^ is 334, and there are 21 particles larger than 50 μm^2^. It should be mentioned that the larger areas of Pb are easily dislodged from the α-Cu matrix during the friction process, which is unfavorable to the tribological performance [5]. Figure 5 b–e show that Bi particles are diffusely distributed on the surface of the Cu matrix, and with the increase in Bi content, the number of Bi particles in the range of 0–10 μm^2^ is also gradually increased, and the fine-sized Bi particles are more conducive to the friction-reducing property of alloy [23].

### 3.2. Properties

**Figure 6:**
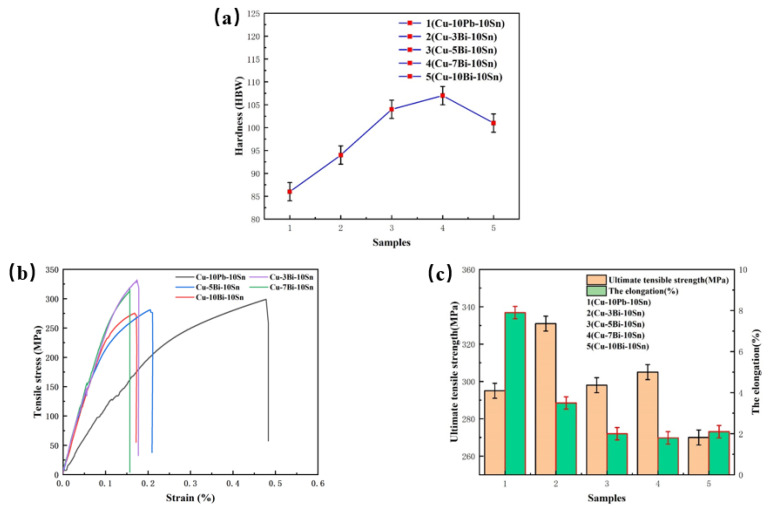
Mechanical properties of the alloys: (**a**) hardness (**b**) tensile stress–strain curves and (**c**) tensile strength and elongation.

The mechanical properties of the alloys are shown in Figure 6. It can be seen that the hardness of the Cu-xBi-10Sn alloy is higher than that of the Cu-10Pb-10Sn alloy, as shown in Figure 6a, and with the increase in Bi content, the hardness of the Cu-xBi-10Sn alloy first increases and then decreases. When the content of Bi is 7 wt.%, the maximum value of 107 HBW is obtained. In this case, the hardness of Cu-7Bi-10Sn alloy is increased by 24.4% compared with that of the Cu-10Pb-10Sn alloy. As mentioned above in Figure 3 and Figure 5, the Bi phase is small and numerous compared with the Pb phase and is diffusely distributed on the substrate. Therefore, it is deduced that a small addition of Bi can achieve the effect of dispersion strengthening, contributing to a higher hardness of the Cu-Bi-Sn alloy [24]. On the contrary, however, the Bi phase grows and increases in number when its content exceeds 7 wt.%. Additionally, the Bi phase is commonly regarded as a soft and brittle phase. As a result, the hardness of alloy decreases. Figure 6b shows the stress–strain curves of the Cu-10Pb-10Sn and Cu-xBi-10Sn alloys, from which there is no yield plateau on the curves, and the stress decreases rapidly when it reaches the peak value. This clearly reveals the brittle fracture of the alloys. That is, there is no correlation between hardness and ultimate tensile strength, which could be due to the brittle fracture before reaching the yielding point. Figure 6c provides the results of the tensile strength of the Cu-10Pb-10Sn and Cu-xBi-10Sn alloys. It is obvious that there is a nonlinear relationship between Bi content and the tensile strength of the alloys. With the increase in Bi content, the tensile strength of the Cu-xBi-10Sn alloy shows a decreasing trend; when the Bi content is 3 wt.%, it reaches a maximum value of 331 MPa. When the contents of Bi are 5 wt.% and 7 wt.%, the tensile strength of the alloys is close to that of the Cu-10Pb-10Sn alloy, which is approximately 295 MPa. The elongation of the Cu-xBi-10Sn alloy was significantly reduced by the addition of Bi. As the Bi metal has brittle characteristics, adding Bi into the alloy greatly reduces the plasticity of the alloy, resulting in a significant decrease in elongation [18,25].

**Figure 7:**
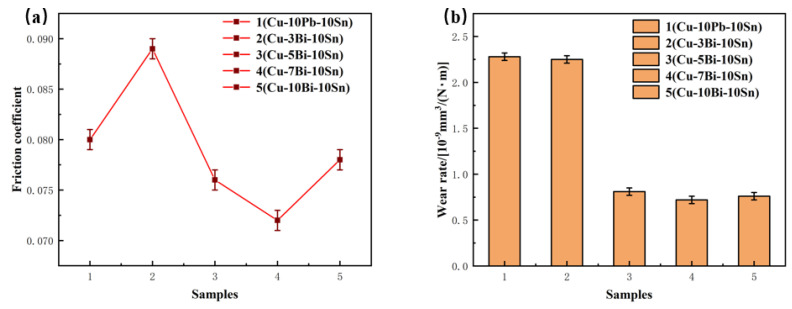
Tribological properties of the alloys: (**a**) average friction coefficient and (**b**) wear rate.

Figure 7 gives the variation curve of friction coefficient and the wear rate of the Cu-10Pb-10Sn and Cu-xBi-10Sn alloys. From Figure 7a, the average friction coefficient of Cu-10Pb-10Sn is 0.08 at a constant speed and constant load, while the Cu-xBi-10Sn alloy shows a trend of firstly decreasing and then increasing with the increase in Bi content. When the content of Bi is 3 wt.%, the average coefficient of friction of the Cu-3Bi-10Sn alloy is 0.089, increasing by 11.25%. The coefficient of friction fluctuates in the range of 0.07 to 0.08 when Bi content is above 5 wt.%, and the average friction coefficient was as low as 0.072 when the Bi content reached 7 wt.%. During the wear test, Bi and Pb as low melting point lubrication components will melt and precipitate on the contact surface of the friction pair due to frictional heating, reducing the shear strength of the friction pair interface. Under the action of friction, Bi and Pb will diffuse into the contact point and form a lubricating film on the contact surface, avoiding direct contact of the materials, and resisting wear and abrasion, thus reducing the coefficient of friction of the friction pair [26,27]. The wear rate is shown in Figure 7b. It can be seen that with the increase in Bi content, the trend of the wear rate is similar to that of the friction coefficient, and the wear rate reaches a minimum value of 0.72 when the Bi content is 7 wt.%. However, the friction coefficient and wear rate of the Cu-xBi-10Sn alloy increase significantly with the further increase in Bi content.

### 3.3. Analysis of Friction and Wear Mechanism

**Figure 8:**
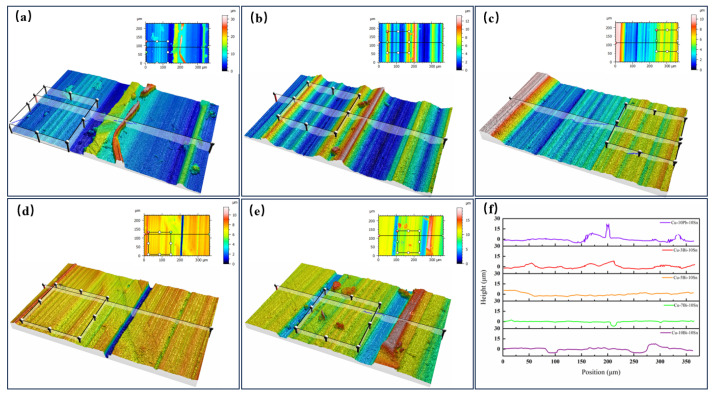
Three-dimensional contours of friction surfaces of the alloys: (**a**) Cu-10Pb-10Sn, (**b**) Cu-3Bi-10Sn, (**c**) Cu-5Bi-10Sn, (**d**) Cu-7Bi-10Sn, (**e**) Cu-10Bi-10Sn and (**f**) cross-section profiles of the Cu-10Pb-10Sn and Cu-xBi-10Sn.

The 3D contours of the worn surfaces with a 365 μm × 288 μm area for the Cu-10Pb-10Sn and Cu-xBi-10Sn alloys are shown in Figure 8. It should be mentioned that the region close to blue has the lowest Z-value and the region close to red has the highest Z-value. The region color can be used to reflect the fluctuation intensity of the alloy’s friction surface profile [28]. From Figure 8, it is revealed that the fluctuation intensity in the Cu-7Bi-10Sn alloy is weaker than that in the other four alloys, which can be characterized by a smaller percentage of blue region on its friction surface, and which usually corresponds to a lower frictional dissipation. Figure 8f shows the cross-section profiles of the Cu-10Pb-10Sn and Cu-xBi-10Sn alloys, which reflect the depth of the alloy abrasion marks more intuitively. It is clear to see that the Cu-7Bi-10Sn alloy has the shallowest abrasion marks, which is consistent with the trend of the friction surface three-dimensional profile changes.

**Figure 9:**
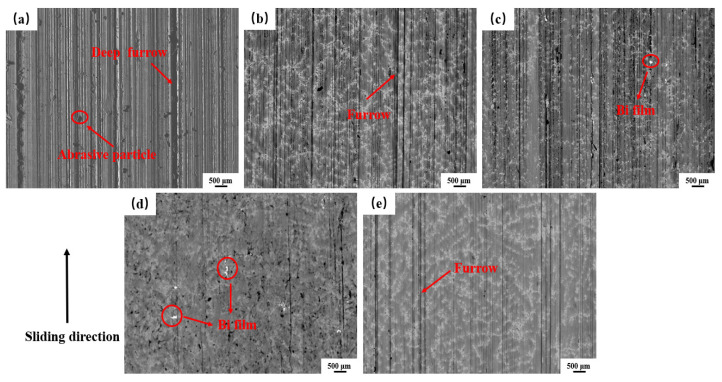
Micrographs of worn surface of the alloys: (**a**) Cu-10Pb-10Sn, (**b**) Cu-3Bi-10Sn, (**c**) Cu-5Bi-10Sn, (**d**) Cu-7Bi-10Sn and (**e**) Cu-10Bi-10Sn.

**Figure 10:**
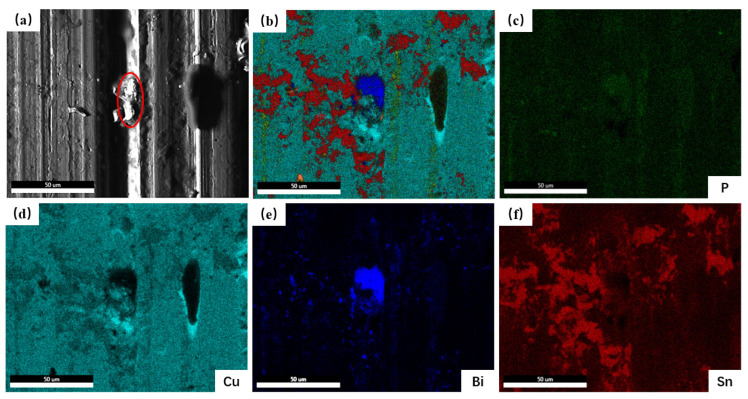
Distribution of elements in the Cu-7Bi-10Sn alloy’s worn surface: (**a**) BSE of Cu-7Bi-10Sn alloy, (**b**) EDS mapping, (**c**) P, (**d**) Cu, (**e**) Bi and (**f**) Sn.

Figure 9 presents the worn surface morphology of the Cu-10Pb-10Sn and Cu-xBi-10Sn alloys. From Figure 9a, the deeper and dense furrows are clearly observed on the worn surface of the Cu-10Pb-10Sn alloy, and the abrasive particle is also present in the localized areas. This is due to the microscopic cutting of softer surfaces by hard particle micro-convexities during the sliding process, resulting in furrows and scratches on the material [29]. Meanwhile, this reflects the fact that the wear mechanism in terms of the Cu-10Pb-10Sn alloy is dominated by abrasive wear. Furthermore, Pb particles are not detected from the worn surface, indicating that the Pb film on the worn surface was worn off. From Figure 9b–e, there are the furrows that exist on the worn surface of the Cu-xBi-10Sn alloys. That is, the addition of Bi has not changed the wear mechanism of the alloys, and the abrasive wear is still dominant. But the influence of Bi content on the degree of wear as it regards the Cu-xBi-10Sn alloys is significantly different. From Figure 9b, although there are still deep furrows on the worn surface, the furrowing effect of the hard particles on the alloy surface is weakened. With the increase in Bi content, as shown in Figure 9c,d, the localized lubricant film rupture, the interaction of the two contact surfaces, brings the local temperature to the flash point, as a low melting point lubrication component, Bi, melts and precipitates out to form a Bi film on the contact surface of the friction pair under the action of friction heat; the Bi film and lubricant work synergistically to reduce the shear strength of the friction interface. However, the excess Bi also affects the tribological properties of the Cu-xBi-10Sn alloys. From Figure 9e, a large number of deep and long furrows appear on the worn surface of the alloy. It indicates that with excessive Bi addition, the frictional stresses cause cracks to be generated and extended in the Bi phase-enriched area, resulting in the detachment of the brittle Bi phase from the Cu matrix during friction and wear tests. The Bi soft lubrication film on the worn surface was also damaged and friction stability was lost. Consequently, the coefficient of friction and the wear rate increased. Figure 10 provides the elements distribution results of the worn surface of the Cu-7Bi-10Sn alloy. It can be seen that the elements of Sn and P are distributed around Bi phase, and it shows that the friction process also plays a role in reducing wear [30,31,32].

The above results indicate that the wear mechanisms of the Cu-10Pb-10Sn alloy and Cu-xBi-10Sn are mainly dominated by abrasive wear. The Cu-10Pb-10Sn alloy’s friction surface has deep furrows and is surrounded by abrasive particles. When the micro-convex body is pressed into the surface of the soft material to a critical depth, the tensile stresses associated with indentation can cause cracks at the bottom of the hard particle micro-convex body. When cracks continue to lengthen and intersect or extend to the surface of hard particles, hard particles are dislodged from the material to form abrasive chips [29]. When the content of Bi is 3 wt.%, the frictional heat generated by the friction partner cannot cause Bi to melt and precipitate, hard particles of micro-convex bodies on the surface of the material are deformed and fractured under the action of shear force and, embedded in the two contact surfaces of the friction vice, abrasive wear occurs on the specimen under the action of friction, resulting in deep furrows on the surface of the alloy [33]. When the content of Bi is 5 wt.% and 7 wt.%, Bi melts and precipitates to form a layer of Bi film on the friction surface, which acts synergistically with the lubricant film to play a good role in reducing wear. When the content of Bi is 10 wt.%, the brittle Bi phase is shed from the friction surface, which in turn intensifies the abrasive wear process. Therefore, the Cu-7Bi-10Sn alloy with 7 wt.% Bi displayed the best friction and wear properties.

## 4. Conclusions

The effects of Bi on the microstructure, mechanical property and wear properties of the tin bronze alloy under oil lubrication at a constant speed and constant load was studied. The mechanism of antifriction and wear resistance of the Bi element was also analyzed. The main conclusions are as follows.

(1) Bi does not generate any compounds with other elements, and it distributes into the bands or long strips on the dendritic arms.
(2) With the increase in the Bi content, the hardness and tensile strength of the Cu-xBi-10Sn alloy show a trend of increasing and then decreasing. When the content of Bi is 7 wt.%, the hardness of the alloy reaches the maximum value of 107 HBW and the tensile strength is close to that of Cu-10Pb-10Sn or 304.5 MPa. The elongation of the alloy is extremely low after adding Bi to tin bronze.
(3) The Cu-xBi-10Sn alloy showed better tribological properties than the Cu-10Pb-10Sn alloy during the wear test under oil lubrication conditions. At a constant speed and load, the average friction coefficient and wear rate of bismuth–tin bronze with a 7 wt.% Bi content are significantly lower than those of lead–tin bronze with a 10 wt.% Pb content. The element Bi exhibits better friction reduction, wear resistance and antifriction properties than Pb.

## Figures and Tables

**Table 1:** Chemical compositions of Cu-10Pb-10Sn/Cu-xBi-10Sn alloys (wt.%).

Alloys	Pb	Bi	Sn	Cu
No.1	10	0	10	Bal.
No.2	0	3	10	Bal.
No.3	0	5	10	Bal.
No.4	0	7	10	Bal.
No.5	0	10	10	Bal.

## Data Availability

Not applicable.

## References

[1] Chen K., Wu X., Zhang A., Zhang J., Chen X., Zhu Y., Wang Z. (2022). Development of wear resistant Cu-12Sn-1.5Ni alloy via minor addition of Fe during casting process. Appl. Surf. Sci..

[2] Turhan H. (2004). Adhesive wear resistance of Cu–Sn–Zn–Pb bronze with additions of Fe, Mn and P. Mater. Lett..

[3] Ren X., Zhang G., Xu H., Wang Z., Liu Y. (2022). Wear Resistance Mechanism of Sub-Nano Cu3P Phase Enhanced the Cu-Pb-Sn Alloy. Coatings.

[4] Ren X., Zhang G., Xu H., Wang Z., Liu Y., Sun F., Kang Y., Wang M., Lv W., Yin Z. (2021). Effects of B on the Structure and Properties of Lead-Tin Bronze Alloy and the Mechanism of Strengthening and Toughening. Materials.

[5] Wang Z., Zhang G., Kang Y., Liu Y., Ren X. (2022). The Effect of Y on the Microstructure, Mechanical and Wear Properties of ZCuSn10Pb10 Alloy. Materials.

[6] Oksanen V.T., Lehtovaara A.J., Kallio M.H. (2017). Load capacity of lubricated bismuth bronze bimetal bearing under elliptical sliding motion. Wear.

[7] Prasad B.K., Patwardhan A.K., Yegneswaran A.H. (2016). Factors controlling dry sliding wear behaviour of a leaded tin bronze. Mater. Sci. Technol..

[8] Ruusila V., Nyyssönen T., Kallio M., Vuorinen P., Lehtovaara A., Valtonen K., Kuokkala V.-T. (2013). The effect of microstructure and lead content on the tribological properties of bearing alloys. Proc. Inst. Mech. Eng. Part J J. Eng. Tribol..

[9] Equey S., Houriet A., Mischler S. (2011). Wear and frictional mechanisms of copper-based bearing alloys. Wear.

[10] Marke K., Petri V., Elena F., Oscar M., Viivi R., Tuomo N., Tapani K.V., Arto L. (2012). Tribological Behavior of Bronze Alloys with Solid Lubricants. Key Eng. Mater..

[11] Thomson J., Zavadil R., Sahoo M., Dadouche A., Dmochowski W., Conlon M. (2010). Development of a lead-free bearing material for aerospace applications. Int. J. Met. Lead. Transf. Res. Technol. Glob. Met. Ind..

[12] Uecker A. (2003). Lead-free carbon brushes for automotive starters. Wear.

[13] Watanabe Y. (2008). High-speed sliding characteristics of Cu–Sn-based composite materials containing lamellar solid lubricants by contact resistance studies. Wear Int. J. Sci. Technol. Frict. Lubr. Wear.

[14] Anton S., Sin D.O.C.V.O., Edvard S.O.C., Smolar T.O.Z. (2002). Process for the Manufacture of a Free-Cutting Aluminum Alloy. U.S. Patent.

[15] Faltus J., Stulíková I., Hájek M., Mádl J., Koutny V., Pla?Ek K., Sláma P. (2002). Aluminium Alloys on the Basis of Al-Cu-Mg, Lead-Free, Intended for Cutting. Mater. Sci. Forum.

[16] Singh R.C., Chaudhary R., Sharma V.K. (2019). Fabrication and sliding wear behavior of some lead-free bearing materials. Mater. Res. Express.

[17] Sircar S. (1997). Free Machining Aluminum Alloy with High Melting Point Machining Constituent and Method of Use. U.S. Patent.

[18] Yin Y., Lin F. (2010). Study on Tribological Properties of Lead-Free Copper-Bismuth Bearing Materials. Met. Funct. Mater..

[19] Chen K., Zhang J., Chen X., Wang Z., Shi R., Zhang A. (2020). The effect of iron on the microstructure and mechanical properties of a cast Cu–12Sn-1.5Ni (wt. %) alloy. Mater. Sci. Eng. A.

[20] Li Z., Chen K., Chen X., Zhu Y., Chen M., Wang Y., Shen J., Shi J., Wang Z. (2022). In-Situ Fabrication, Microstructure and Mechanical Performance of Nano Iron-Rich Precipitate Reinforced Cu and Cu Alloys. Metals.

[21] Fontanari V., Benedetti M., Straffelini G., Girardi C., Giordanino L. (2013). Tribological behavior of the bronze–steel pair for worm gearing. Wear.

[22] Stella J., Gerke L., Pohl M. (2013). Study of cavitation erosion and adhesive wear in CuSnNi alloys produced by different casting processes. Wear.

[23] Yin Y., Li R., Zhang G., Zhang K., Chen Q. (2019). Tribological properties of FeS/Cu-Bi copper-based bearing materials fabricated by mechanical alloying. Ind. Lubr. Tribol..

[24] Shen Y.-A. (2023). Bi Dispersion Hardening in Sn-Bi Alloys by Solid-State Aging. JOM.

[25] Shen Y.-A., Zhou S., Li J., Yang C.-h., Huang S., Lin S.-k., Nishikawa H. (2019). Sn-3.0Ag-0.5Cu/Sn-58Bi composite solder joint assembled using a low-temperature reflow process for PoP technology. Mater. Des..

[26] Park H.I., Park S.I., Kim S.G. (2012). Influence of Bismuth and Antimony Additions on the Structures and Casting Properties of Lead-free Cu-Zn-Sn Bronze Castings. J. Korea Foundry Soc..

[27] Yokota H., Desaki T., Hayakawa H., Hashizume K., Suzuki M. (2006). Newly Development Lead Free Copper Alloy Bushing for Fuel Injection Pump.

[28] Dong B.W., Wang S.H., Dong Z.Z., Jie J.C., Wang T.M., Li T.J. (2020). Novel insight into dry sliding behavior of Cu-Pb-Sn in-situ composite with secondary phase in different morphology. J. Mater. Sci. Technol..

[29] Shaik M.A., Golla B.R. (2019). Development of highly wear resistant Cu-Al alloys processed via powder metallurgy. Tribol. Int..

[30] Bharatish A., Harish V., Bathe R.N., Senthilselvan J., Soundarapandian S. (2018). Effect of scanning speed and tin content on the tribological behavior of femtosecond laser textured tin-bronze alloy. Opt. Laser Technol..

[31] Zhang G., Kang Y., Liu Y., Wang Z., Xu H. (2022). Study on tribology of liquid-solid formed CuPb20Sn5/Carbon steel bimetal. Mater. Res. Express.

[32] Ünlü B.S. (2009). Investigation of tribological and mechanical properties of metal bearings. Bull. Mater. Sci..

[33] Fan J., Zhang C., Wu S., Jia D., Sun L., Li Y., Liu J. (2018). Effect of Cr–Fe on friction and wear properties of Cu-based friction material. Mater. Sci. Technol..

